# Antioxidant, Lipoxygenase and Histone Deacetylase Inhibitory Activities of *Acridocarbus orientalis* From Al Ain and Oman

**DOI:** 10.3390/molecules171112521

**Published:** 2012-10-24

**Authors:** Taoufik Ksiksi, Alaaeldin A. Hamza

**Affiliations:** 1 Department of Biology, Faculty of Science, U.A.E. University, Al-Ain, P.O. Box 17551, UAE; Email: allaaeldin.hamza@uaeu.ac.ae; 2 National Organization of Drug Control and Research, 6 Abu Hazem St., Giza, 12613, Egypt

**Keywords:** *Acridocarpus orientalis*, total phenol, antioxidant, anti-lipoxygenase, anti-histone deacetylase activity

## Abstract

*Acridocarpus orientalis* (AO) is a traditional medicinal plant used for treatment of inflammatory diseases that may have potential in cancer treatment. In the present study, the aqueous ethanolic crude extract of *Acridocarpus* aerial parts obtained from Al Ain and Oman were evaluated for their antioxidant capability, polyphenolic content, anti-lipoxygenase and anti-histone deacetylase (HDAC) properties. The total antioxidant capacity was estimated by the FRAP, DPPH, ABTS and β-carotene bleaching assays. *Acridocarpus*-Al Ain exhibited the highest polyphenolic content (184.24 mg gallic acid/g) and the best antioxidant activity (1.1, 1.04, 1.14 mmol ascorbic acid equivalent/g in the FRAP, ABTS and DPPH assays, respectively). Additionally, the same extract showed significant anti-inflammatory properties via lipoxygenase (LOX) inhibitory activity (IC_50_ = 50.58 µg/mL). *Acridocarpus*-Al Ain also showed the strongest histone deacetylase (HDACs) inhibitory activity (IC_50_ = 93.28 µg/mL). The results reported here suggest that there was a significant influence of location and the plant may be considered a good source of compounds with antioxidant, anti-LOX and HDAC properties for therapeutic, nutraceutical and functional food applications.

## 1. Introduction

Many plant species represent a large source of structurally new compounds that might serve as leads for the development of new drugs, nutraceuticals and functional foods. Much of the therapeutic activity of plants is due to their biologically active polyphenolic substances, mostly flavonoids and phenolic acids, which possess antioxidant, anti-lipoxygenase and anticancer activities [1,2]. In developing countries and particularly the Gulf Countries, large segments of the population still rely on folk medicine to treat serious diseases including cancers and various types of inflammations. 

Reactive oxygen species (ROS) induce oxidative damage to biomolecules like lipids, nucleic acids, proteins and carbohydrates. This damage causes the onset of many diseases such as rheumatoid arthritis, cirrhosis, arteriosclerosis, diabetes and cancer [3]. ROS also affect food quality. Interest in finding naturally occurring antioxidants for use in food preservation, flavoring, cosmetics, and in health promotion to replace synthetic antioxidants, that are being restricted due to their carcinogenicity [2] has increased noticeably. In addition, ROS propagate inflammation by stimulating release of cytokines and activation of enzymes such as lipoxygenases (LOXs) from inflammatory cells. LOXs are the key enzymes in the biosynthesis of leukotrienes from fatty acids producing active lipid metabolites. LOX is involved in provoking several inflammation-related diseases such as arthritis, asthma, cardiovascular, cancer and allergic diseases [4,5]. For this reason, targeting inhibitors of LOX is a promising therapeutic target for treating wide spectrum of human diseases. 

Histone deacetylases (HDACs) are becoming a prominent therapeutic target for treatment of cancer and other diseases [6]. HDAC inhibitors (HDACI) represent a novel class of targeted drugs which alter the acetylation statues of several proteins. These agents, modulating both chromatin structure through histone acetylation, and the activity of several non-histone substrates, are able to determine changes in gene transcription and to induce a plethora of biological effects ranging from cell death induction, to angiogenesis inhibition or modulation of immune responses [7]. The shortcomings of HDACs are instability and toxicity [8,9]. For this reason, targeting natural inhibitors of HDAC is a promising therapeutic target for treating a wide spectrum of human diseases

*Acridocarpus orientalis* (AO), belonging to the Malpighiaceae family, is widespread in tropical Africa, Asia and the Mediterranean region and in the sandy plains in the Western Gulf countries. Currently, it is being cultivated in the greenhouse and under laboratory conditions [10]. *Acridocarpus socotranus* is commonly traditionally used in Yemen for the treatment of headaches and muscle pain [11,12]. The leaves and bark of *Acridocarpus chloropterus* in Tanzania have been reported to have antiplasmodial, anti-trypanosomal and anti-leishmanial activities [13]. Several species of *Acridocarpus* are still used traditionally all over the World as folk medicines, and more specific research to justify this is essential.

Based on the above rationale, the objective of this research focused on the quantitative determination of the phenolic content, antioxidant, anti-lipoxygenase and anti-HDAC activities of the aqueous ethanol extract of AO obtained from Al Ain and Oman. The antioxidant potential of AO was assessed in comparison with the scavenging power of the two stable nitrogen–centered radicals, 1,1-diphenyl-2-picrylhydrazyl (DPPH•) and 2,2-azino-bis(3-ethylbenzothiazoline-6-sulfonate) radical (ABTS^•+^). The reducing power of antioxidants was assessed by the ferric reducing antioxidant power (FRAP) assay as well as anti-bleaching of β-carotene activity. LOX inhibitory activity of *Acridocarpus* extracts was also measured. Finally, HDAC inhibition activity of *Acridocarpus* extracts was measured with a HDAC Colorimetric Assay Kit (Millipore Corporation).

## 2. Results and Discussion

### 2.1. Antioxidant, Free Radical Scavenging Activity

One single method cannot precisely assess the antioxidant capacities of plant extracts due to the complex nature of the different phytochemical classes present in plants. In the present work, the FRAP, ABTS, DPPH• and β-carotene assays were used to test the antioxidant activities of *Acridocarpus orientalis* extracts. The results of the four assays are summarized in Table 1.

**Table 1:** Total antioxidant activity of ethanol extract from *Acridocarpus orientalis* expressed as ascorbic acid equivalents (mmol/g of dry extract). Trolox was used as positive control.

Extract	FRAP Assay	ABTS Assay		DPPH Assay		β-Carotene Assay
	TAC (mmol/g)	TAC (mmol/g)	IC_50_ (µg/mL)	TAC (mmol/g)	IC_50_ (µg/mL)	IC_50_ (µg/mL)
***Acridocarpus*-Al Ain (a)**	1.10 ± 0.01	1.04 ± 0.02	58.06 ± 1.39 ^b,c^	1.14 ± 0.01	29.84 ± 0.59 ^b,c^	5.0 ± 0.04 ^b,c^
***Acridocarpus*-Oman (b)**	0.96 ± 0.02	0.98 ± 0.01	72.32 ± 1.61 ^a,c^	1.04 ± 0.36	32.44 ± 0.34 ^a,c^	6.32 ± 0.04 ^a,c^
**Trolox (c)**	3.86 ± 0.09 ^a,b^	1.83 ± 0.45	33.58 ± 1.43 ^a,b^	3.99 ± 0.03	10.07 ± 0.09 ^a,b^	3.03 ± 0.04 ^a,b^

Values are means ± SE of three experiments. Data with on letters are significantly different (*p* < 0.05).

#### 2.1.1. FRAP Assay

FRAP assay depends on reduction of oxidized ferric ions to ferrous ions by antioxidant agents. Table 1 shows that all extracts exhibited some degree of electron donation capacity. Extract of *Acridocarpus*-Al Ain exhibited the highest antioxidant potency (1.10 mmol ascorbic acid equivalent/g). The antioxidant potency of in FRAP assay was in the following order Trolox (3.86 mmol/g) > *Acridocarpus*-Al Ain extract (1.10 mmol/g) > *Acridocarpus*-Oman extract (0.96 mmol/g). The FRAP values of *Acridocarpus* extracts were much higher than those values reported for other plants such as *Leptadenia pyrotechnica* Forssk (0.24 mmol/g) [14].

#### 2.1.2. ABTS Radical Scavenging Assay

**Figure 1:**
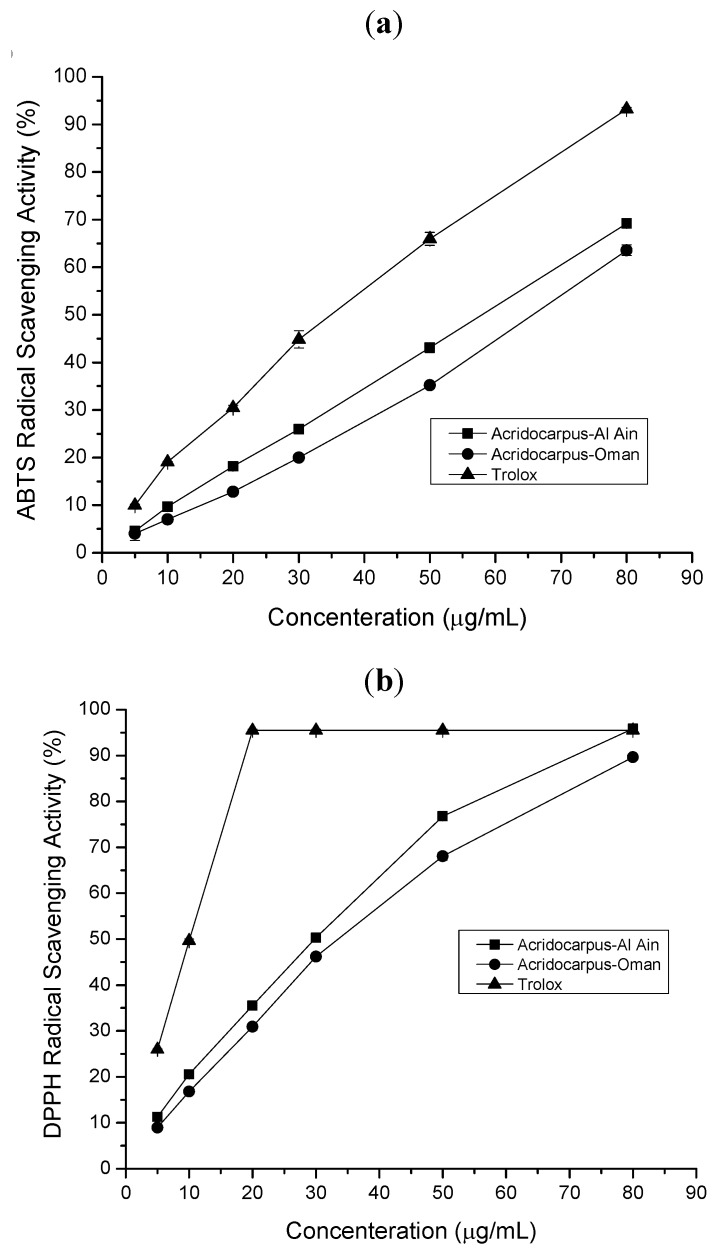
(**a**) ABTS and (**b**) DPPH radical scavenging activities of *Acridocarpus* ethanol extracts and Trolox at various concentrations. Values are means ± SE of three experiments.

ABTS assay expressed as ascorbic acid equivalent/g dry extract varied from 1.83 mmol/g for Trolox to 0.98 mmol/g dry extract for *Acridocarpus*-Oman extract. In addition, the antioxidant activity evaluated as IC_50_ value (µg/mL) revealed a similar behavior. The best free radical scavenging activities are reflected by the smallest IC_50_ values. The percentage inhibition of ABTS radical scavenging ability of two *Acridocarpus* extracts was concentration dependent (Figure 1a). The *Acridocarpus*-Al Ain extract showed the strongest activity with IC_50_ = 58.06 µg/mL which was better than *Acridocarpus*-Oman extract with IC_50_ = 72.32 µg/mL. It is also important to note that the ABTS radical scavenging ability of *Acridocarpus* extracts was much greater than those values reported for peanuts (81.3 µmol/g) and pistachios (75.9 µmol/g) [15].

#### 2.1.3. DPPH Radical Scavenging Assay

The DPPH radical- scavenging assay is a widely used method for evaluating the ability of plant extracts to scavenge free radical generated from DPPH reagent. DPPH, a stable free radical with purple color, changes into a stable yellow compound on reacting with an antioxidant. The DPPH radical is scavenged by antioxidants through donation of hydrogen radicals (H•) to form the stable DPPH-H molecule [16]. The results of the DPPH assay are shown in Table 1. The total antioxidant activity expressed as ascorbic acid equivalent/g dry extract varied from 3.99 mmol/g for Trolox to 1.04 mmol/g dry extract for *Acridocarpus*-Oman extract. In addition, the DPPH radical scavenging ability of samples evaluated as IC_50_ value (µg/mL). The best free radical scavenging activities (reflected by the smallest IC_50_ values) was exerted by *Acridocarpus*-Al Ain (IC_50_ = 29.84 µg/mL), which contained the highest amount of total phenolic. The lowest radical scavenging activity was exhibited by *Acridocarpus*-Oman extract (IC_50_ = 32.44 µg/mL). Scavenging abilities of the extracts were in the following order Trolox > *Acridocarpus*-Al Ain extract > *Acridocarpus*-Oman extract. The percentage inhibition DPPH radical scavenging ability of two *Acridocarpus* extracts was concentration dependent (Figure 1b). The DPPH radical-scavenging activity of *Acridocarpus* ethanol extracts was relatively greater than those reported for other plant species such as basil (IC_50_ = 500 µg/mL), sage (IC_50_ = 400 µg/mL) thyme (IC_50_ = 470 µg/mL), oregano (IC_50_ = 320 µg/mL), rosemary (IC_50_ = 180 µg/mL) and fennel (IC_50_ = 148 µg/mL) [17].

#### 2.1.4. β-Carotene Bleaching Test

β-Carotene undergoes rapid bleaching in the absence of antioxidants. The presence of antioxidants hinders the extent of bleaching by neutralizing the linolic hydroperoxyl radical formed. The Trolox (IC_50_ = 3.03 µg/mL) and *Acridocarpus*-Al Ain extract (IC_50_ = 5.0) showed the highest ability to prevent bleaching of β-carotene, followed by *Acridocarpus*-Oman extract (IC_50_ = 6.32 µg/mL). The inhibition of bleaching of β-carotene capacity of *Acridocarpus* ethanol extract was substantially higher than those reported for mint and radish (IC_50_ > 100 µg/mL) [18] and chicory (IC_50_ > 100 µg/mL) [19]. These results led us to conclude that the antioxidant compounds extracted from *Acridocarpus orientalis* are more concentrated in Al Ain. 

### 2.2. Total Phenolic Content

The total phenolic content of the medical plants extracts was measured with the Folin-Ciocalteu reagent assay and the results is shown in Table 2. The values varied from 184.24 to 149.23 mg gallic acid/g of dry extract. *Acridocarpus*-Al Ain exhibited the highest amount of total phenolics (184.24 mg gallic acid equivalent/g). Phytochemicals have been of great interest as a source of natural antioxidants used for health promotion, food preservation, food flavoring and cosmetics since they are safer for consumption and more environmentally friendly than their synthetic counterparts.

**Table 2:** Total phenolic content of ethanol extract from *Acridocarpus orientalis* expressed as gallic acid equivalents (mg/g of dry extract).

Extract	Yield (%)	Total Phenolic Content * (mg/g)
*Acridocarpus*-Al Ain (a)	30%	184.24 ± 4.39 ^b^
*Acridocarpus*-Oman (b)	22%	149.23 ± 2.84 ^a^

* Values are means ± SE of three experiments. Data with on letters are significantly different (*p* < 0.05).

### 2.3. Correlation between Antioxidant Activity and Phenolic Contents

The linear correlation coefficients between the antioxidant capacity (measured by FRAP, DPPH, ABTS and β-carotene assays and total phenolic content of the four fractions are shown in Table 3. R^2^ values varied between 0.99 for β-carotene assay and 0.87 DPPH assays, which leads to believe that the presence of phenolic compounds contributes substantially to the antioxidant activity of the tested extracts, especially in the β-carotene assay. The above results are in agreement with previous reports that showed a liner correlation between the total phenolic content and the reducing antioxidant capacity of some plant extracts [16,20].

**Table 3:** Linear correlations between the amount of total phenolic content and antioxidant activities of ethanol extract from *Acridocarpus orientalis*.

Assay	Correlation (R^2^)	Significance
FRAP activity	0.93	*p* < 0.01
ABTS• scavenging activity	0.94	*p* < 0.01
DPPH• scavenging activity	0.87	*p* < 0.01
β-Carotene bleaching inhibition	0.99	*p* < 0.001

### 2.4. LOX Inhibitory Assay

**Figure 2:**
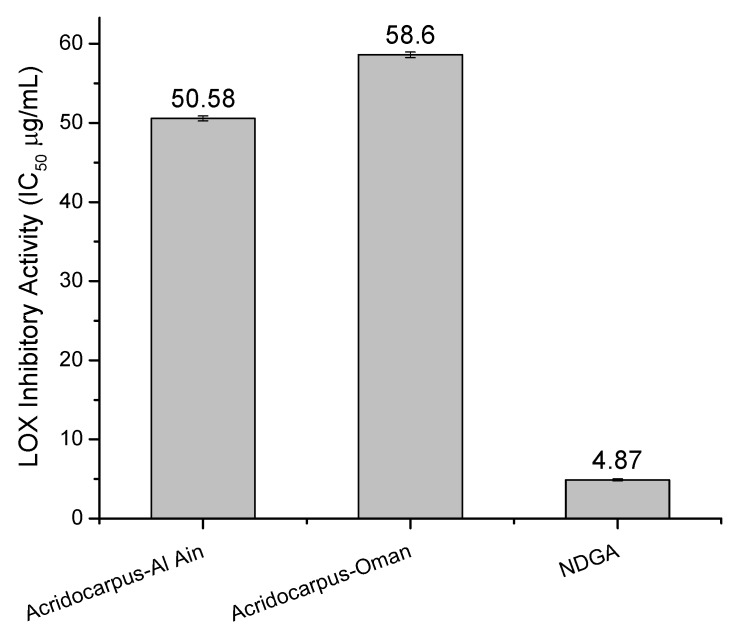
LOX inhibitory activity of *Acridocarpus orientalis* extracts expressed as IC_50_ (µg/mL). Values are means ± SE of three experiments.

LOX catalyzes dioxygenation of polyunsaturated fatty acids to yield *cis,trans*-conjugated diene hydroperoxides. Results for LOX inhibitory activity (IC_50_) are shown in Figure 2. The *Acridocarpus*-Al Ain extract showed a strongest ability (*p* < 0.05) to inhibit LOX activity (IC_50_ = 50.58 µg/mL) in relation to the *Acridocarpus*-Oman extract (IC_50_ = 58.61 µg/mL). It is important to note that that both crude ethanol extracts possessed significantly lower (*p* < 0.05) LOX inhibitory activity than that of an NDGA positive standard (IC_50_ = 4.87 µg/mL). Finally, LOX inhibition of *Acridocarpus orientalis* extracts were higher than those for other common plants such as *Thespesia lampas* (600 µg/mL) [21]. The results reported here suggest that *Acridocarpus orientalis* has potentially high anti-LOX effect, which might be related to the polyphenolic content and antioxidant property of the extract.

### 2.5. HDAC Inhibitory Assay

**Figure 3:**
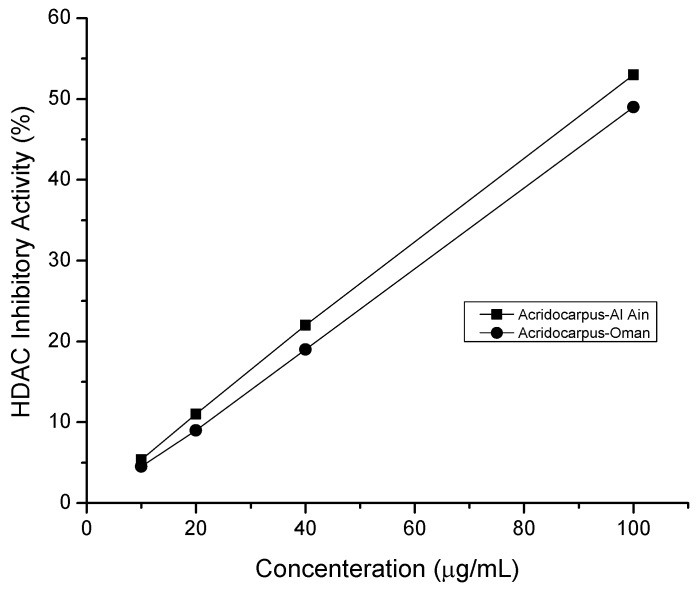
Concentration-response curve of HDAC inhibitory activity by *Acridocarpus*-Al Ain (IC_50_ = 93.28 µg/mL) and *Acridocarpus*-Oman (IC_50_ = 102.5 µg/mL).

HDAC inhibitors (HDACi) are becoming a prominent therapeutic target for treating a wide spectrum of human diseases, including cancer. HDAC inhibitors arrest cell growth and lead to apoptosis in tumor cells [6]. In previous studies, Cardiotoxicity, poor pharmacokinetic and non-selectivity were repeatedly reported for different HDACi and considered as the major drawback for the future development of synthetic HDACi [8,22]. For this reason, targeting natural inhibitors of HDAC is a promising therapeutic target for treating wide spectrum of human diseases. Curcumin, a phenolic compound isolated from turmeric, and sulforaphane, an isothiocyanate isolated from broccoli, have recently been identified to possess anticancer properties via HDACi activity [22,23]. In the present work, the ethanolic extract of *Acridocarpus orientalis* showed a high ability to inhibit HDAC activity (IC_50_ = 93.28 µg/mL) in Al Ain species in relation to the Oman plant extract (IC_50_ = 102.5 µg/mL) and this inhibitory effect was concentration dependent (Figure 3). This inhibitory effect of plant extract could be attributed to a direct ionic interaction of the active ingredients of plant with the active zinc site of HDAC enzyme where the classical HDACs are zinc-dependent enzymes bearing a highly conserved catalytic domain with a zinc ion [6].

## 3. Experimental

### 3.1. Chemicals

Ascorbic acid, ferric chloride, Folin-Ciocalteu reagent, dibutylhydroxytoluene (BHT), 2,4,6-tripyridyltriazine, gallic acid, sodium carbonate, 1,1-diphenyl-2-picrylhydrazyl (DPPH) and 2,4,6-tripyridyl triazine, 2,2-azino-bis(3-ethylbenzothiazoline-6-sulfonate) (ABTS), β-carotene (type I ≥ 93%) Trolox, soybean LOX and NDGA were obtained from Sigma Chemical Co. (St. Louis, MO, USA). HDAC colorimetric assay kit was purchased from Millipore Corporation (Temecula, CA, USA). All other chemicals were purchased from local commercial suppliers.

### 3.2. Plant Materials

Fresh *Acridocarpus orientalis* samples were collected from a dry river, on Jebel Hafeet mountain near Al Ain, UAE (N 24.19, E 55.62) and from Nizwa mountain in Oman (N22.9, E57.5). The collected samples were identified and representative specimens were deposited at the Biology Department Herbarium, Faculty of Science, UAE University.

### 3.3. Preparation of the Plant Extracts

An air-dried and ground aerial part sample of AO (10 g) was extracted with 70% (v/v) aqueous ethanol (200 mL). The mixture was macerated for 72 h at 4 °C. The resulting compound was then filter dried under reduced pressure in a rotary evaporator at 40 °C. An aqueous ethanol crude extract was generated. This crude extract was weighed, dissolved in 50% ethanol (typically 30 mg/mL) and kept at −20 °C for further analysis.

### 3.4. Total Phenolic Content Determination

Total phenolic content was assessed using the method reported by Singleton *et al*. [24] using the Folin-Ciocalteu reagent. For a typical plant extract, a sample of the residue obtained from the crude extract described in the section above (100 µL) was thoroughly mixed with the Folin-Ciocalteu reagent (200 µL) and de-ionized water (2 mL). An incubation period at room temperature for 3 min followed. After incubation, a sample of 20% aqueous sodium carbonate (w/w, 1 mL) was added to the mixture. After one hour of incubation at room temperature, the total polyphenols were determined by measuring the absorbance of the resulting substance at 765 nm with a PerkinElmer, Lambda 25 UV/VIS spectrophotometer. Values were expressed in milligrams of gallic acid equivalent per gram dry weight of plant species extract. Data presented here are averages of three replicates.

### 3.5. Estimation of Total Antioxidant Activity

#### 3.5.1. FRAP assay

The reducing power of antioxidants by the decrease of the ferric ions to the ferrous ions constitutes the basis of the FRAP assay. A blue colored ferrous-tripyridyltriazine complex is formed, as per the method reported by Nenadis *et al*. [25]. The FRAP reagent was freshly prepared by mixing 2,4,6-tripyridyltriazine (10 mM, 1.0 mL) and ferric chloride (20 mM, 1.0 mL) in acetate buffer (0.25 M, 10 mL, pH 3.6). Samples of plant species extract (50 µL) were added to the FRAP reagent (3.0 mL). The tests were carried out in triplicates. After a period of 8 min incubation at room temperature, the absorbance was measured at 593 nm. A calibration curve of ascorbic acid was developed to quantitatively determine the antioxidant capacity of the plant extracts expressed as mmol ascorbic acid equivalent per gram of dry extract.

#### 3.5.2. ABTS Assay

The reduction of the blue-green 2,2-azino-bis(3-ethylbenzothiazoline-6-sulfonate) radical cation (ABTS^•+^) by antioxidants to its original colorless ABTS form is the basis of the ABTS assay. The ABTS^•+^ is decolorized by antioxidants according to their antioxidant content [25]. A mixture of ABTS (10 mmol) and hydrogen peroxide (28.3 µmol) in acetic acid-sodium acetate buffer (30 mmol, pH 3.6, total volume of 2.0 mL) was quickly mixed with the plant species extract or a standard compound (100 µL) in a test tube. For positive referencing, Dibutyl hydroxytoluene (BHT) was used. The content of the tube was mixed and allowed to stand for 6 min and the absorbance was measured at 660 nm. Inhibition of free radical scavenging activity was calculated using the equation:

% Inhibition = 100 × (absorbance of the control − absorbance of the sample)/absorbance of the control

EC_50_ value (µg/mL) is the effective concentration at which ABTS^•+^ is scavenged by 50%. A calibration curve of ascorbic acid was established, the antioxidant content of the plant species extracts were then expressed as mmol ascorbic acid equivalent per gram of dry plant extract.

#### 3.5.3. DPPH• Radical Assay

The DPPH stable radical scavenging capabilities of plant extract was determined according to a standard procedure reported by Nenandis *et al.* [25]. Methanolic solution of DPPH radical (3.8 mL, 60 µg/mL) was quickly mixed with the plant extract (200 µL) in a test tube. BHT was used as a positive standard. The contents of the tube were mixed and rested for 30 min. The absorbance was then measured at 517 nm. Inhibition of free radical scavenging activity was calculated using the equation:

% Inhibition = 100 × (absorbance of the control − absorbance of the sample)/absorbance of the control.

EC_50_ value (µg/mL) is the effective concentration at which DPPH• radicals are scavenged by 50%. A calibration curve of ascorbic acid was developed. The antioxidant content of the plant extracts were expressed as mmol ascorbic acid equivalent per gram of dry extract.

#### 3.5.4. β-Carotene Bleaching Assay

The prevention of β-carotene bleaching by plant extracts was assessed according to the procedure of Lim *et al*. [16]. A sample of β-carotene solution (1.0 mL, 200 µg/mL in chloroform) was mixed with of linoleic acid (200 µL) and Tween 20 as emulsifier (200 µL). The mixture was evaporated to remove chloroform in a rotary evaporator at 40 °C. Deionized water (100 mL) was added slowly to form an emulsion. Portions of β-carotene/linoleic acid emulsion (3 mL each) were mixed in test tubes with 200 µL of various plant extract concentrations. The control was a 200 µL of 50% of methanol in 3.0 mL of the above emulsion. As a positive standard, BHT was used. After an incubation period of 120 minutes at 45 °C, the absorbance of the samples, standards and control were measured at 470 nm. Inhibition of free radical scavenging activity was calculated using the equation:

% Inhibition = 1 − (absorbance of the control at time zero − absorbance of the control after 120 min)/absorbance of the sample at time zero − absorbance of the sample 120 min) × 100

EC_50_ value (the effective concentration at which bleaching of β-carotene is prevented by 50% µg/mL) was determined graphically.

### 3.6. LOX Inhibitory Assay

Lipoxygenase (EC 1.13.11.12 type 1-B) (LOX) was assayed according to the method reported by Wu [26]. A mixture of a solution of sodium borate buffer (1 mL, 0.1 M, pH 8.8) and soybean LOX (10 µL, final conc. 8,000 U/mL) was incubated with plant species extract sample (10 µL) in a 1 mL cuvette at room temperature for 5 min. The reaction was initiated by the addition of linoleic acid substrate (10 µL, 10 mmol). The absorbance of the resulting mixture was measured at 234 nm over time at a rate of one measurement/min (3 readings). Inhibition of LOX was assessed using the following equation:

% Inhibition = 100 × (absorbance of the control − absorbance of the sample)/absorbance of the control)

The effective concentration (µg/mL) at which LOX activity is inhibited by 50% (IC_50_) was represented in a graph. Nordihydroguaiaretic acid (NDGA) was used as a positive standard.

### 3.7. HDAC Inhibition Activity Screening

HDAC inhibitory activity of Acridocarpus extracts was measured with HDAC Colorimetric Assay Kit (Millipore Corporation, Catalog number: 17-374). Plant extracts and trichostatin A, an inhibitor of HDAC, were mixed with Hela nuclear extract that contains a variety of HADC enzymes and has HDAC activity. HDAC colorimetric substrate was added to inhibitor and Hela nuclear extract mixture. A color is developed after a one hour treatment with the lysine developer. Absorbance at 405 was measured by micro plate reader model.

% Inhibition = 100 × (absorbance of the Hela nuclear extract − absorbance of the sample)/absorbance of the Hela nuclear extract)

The effective concentration (µg/mL) at which HDAC activity is inhibited by 50% (IC_50_) was determined graphically.

### 3.8. Statistical Analysis

Reported data are expressed as means ± SEM. Correlation analysis of antioxidants and the total phenolic content was conducted using SPSS (SPSS Inc., Chicago, IL, USA). When significant differences were detected, an analysis of differences between the means was performed by using Tukey’s HSD multiple comparison tests. Significance levels were set at *p* < 0.05.

## 4. Conclusions

The present biological investigation of Acri*docarpus orientalis* crude extracts demonstrated promising antioxidant, anti-LOX and anti-HDAC properties. Presumably, these biological activities could be attributed in part to the polyphenolic features of this plant species. This is absolutely the first published report on the biological properties of *Acridocarpus orientalis* growing in Al Ain and Oman. More *in-vivo* and *in-vitro* studies along with detailed phytochemical investigations are needed in order to potentially use this plant in the prevention and therapies of some oxidative damage related diseases. In short, the present study provides the biochemical foundation for further chemical analysis. Some animal as well as clinical studies are underway in our labs.
