# Erratum: Ksiksi, T., et al. Antioxidant, Lipoxygenase and Histone Deacetylase Inhibitory Activities of *Acridocarpus orientalis* from Al Ain and Oman. *Molecules* 2012, *17*, 12521–12532.

**DOI:** 10.3390/molecules22071090

**Published:** 2017-06-30

**Authors:** Molecules Editorial Office

**Affiliations:** MDPI AG, St. Alban-Anlage 66, 4052 Basel, Switzerland

The Molecules Editorial Office wishes to make the following erratum to this paper [1]. There is a misspelling in the title of “Antioxidant, Lipoxygenase and Histone Deacetylase Inhibitory Activities of *Acridocarbus orientalis* from Al Ain and Oman.” The correct title should be “Antioxidant, Lipoxygenase and Histone Deacetylase Inhibitory Activities of *Acridocarpus orientalis* from Al Ain and Oman.”

We apologize for any inconvenience caused to the readers by this mistake.
